# *CDC73* Intragenic Deletion in Familial Primary Hyperparathyroidism Associated With Parathyroid Carcinoma

**DOI:** 10.1210/jc.2014-1481

**Published:** 2014-05-13

**Authors:** Eeva Korpi-Hyövälti, Treena Cranston, Eeva Ryhänen, Johanna Arola, Kristiina Aittomäki, Timo Sane, Rajesh V. Thakker, Camilla Schalin-Jäntti

**Affiliations:** Department of Internal Medicine, Seinäjoki Central Hospital (E.K.-H.), 60320 Seinäjoki, Finland; Oxford Medical Genetics Laboratory, Churchill Hospital (T.C.), Division of Endocrinology, Department of Medicine (E.R., T.S., C.S-J.), Department of Pathology (J.A.), and Department of Medical Genetics (K.A), Helsinki University Central Hospital, FI-00290 Helsinki, Finland; and Academic Endocrine Unit, Radcliffe Department of Medicine, University of Oxford (R.V.T.), Oxford OX1 2JD, United Kingdom

## Abstract

**Context:**

*CDC73* mutations frequently underlie the hyperparathyroidism-jaw tumor syndrome, familial isolated hyperparathyroidism (FIHP), and parathyroid carcinoma. It has also been suggested that *CDC73* deletion analysis should be performed in those patients without *CDC73* mutations.

**Objective:**

To investigate for *CDC73* deletion in a family with FIHP previously reported not to have *CDC73* mutations.

**Patients and Methods:**

Eleven members (six affected with primary hyperparathyroidism and five unaffected) were ascertained from the family, and multiplex ligation-dependent probe amplification was performed to detect *CDC73* deletion using leukocyte DNA.

**Results:**

A previously unreported deletion of *CDC73* involving exons 1–10 was detected in five affected members and two unaffected members who were 26 and 39 years of age. Two affected members had parathyroid carcinomas at the ages of 18 and 32 years, and they had Ki-67 proliferation indices of 5 and 14.5% and did not express parafibromin, encoded by *CDC73*. Primary hyperparathyroidism in the other affected members was due to adenomas and atypical adenomas, and none had jaw tumors. Two affected members had thoracic aortic aneurysms, which in one member occurred with parathyroid carcinoma and renal cysts.

**Conclusion:**

A previously unreported intragenic deletion of exons 1 to 10 of *CDC73* was detected in a three-generation family with FIHP, due to adenomas, atypical adenomas, and parathyroid carcinomas. In addition, two affected males had thoracic aortic aneurysms, which may represent another associated clinical feature of this disorder.

Primary hyperparathyroidism (PHPT) is a common endocrine disease; the prevalence is 1–4 per 1000, and it increases to 21 per 1000 in age groups 55–75 years (1). In 5–10% of cases, PHPT is part of a genetic syndrome, such as multiple endocrine neoplasia type 1 or 2, hyperparathyroidism-jaw tumor syndrome (HPT-JT), familial isolated hyperparathyroidism (FIHP), or familial hypocalciuric hypercalcemia (1–5). The HPT-JT syndrome is an autosomal dominant disease characterized by parathyroid tumors and ossifying tumors of the jaw (4). Some patients also develop renal and uterine tumors (4). The HPT-JT syndrome is due to mutations of the cell division cycle protein 73 homolog (*CDC73*) gene, located at 1q31.2 (3, 6–8). *CDC73* has 17 exons, acts as a tumor suppressor gene, and encodes parafibromin, a 531-amino acid protein predominantly expressed in the nucleus (4). Parafibromin serves as a parathyroid carcinoma marker because it is expressed in normal parathyroid glands, parathyroid hyperplasia, and adenomas but is usually absent in parathyroid carcinomas (9–11) and occasionally in atypical adenomas. To date, more than 60 *CDC73* germline mutations have been reported, the majority being frameshift, nonsense, and missense mutations (12). Approximately 55% of *CDC73* mutations are associated with HPT-JT and over 20% with FIHP (12). HPT-JT and FIHP patients who do not have *CDC73* mutations may have intragenic or whole deletions of *CDC73* (13–15), and >5% of PHPT patients without *CDC73* mutations have been reported to have large *CDC73* deletions (15). Thus, it is recommended that deletion analysis of the *CDC73* gene should be performed in HPT-JT, FIHP, parathyroid carcinoma, or severe early-onset PHPT patients who do not have *CDC73* point mutations (15–17). We report a three-generation family with FIHP, in whom *CDC73* mutations were not identified in the original report (8), but in whom we have now identified an intragenic deletion involving exons 1 to 10.

## Subjects and Methods

**Figure 1.**
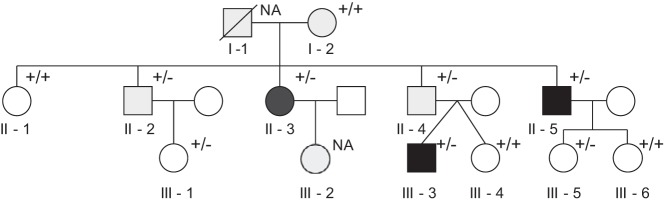
Family tree. White denotes normal biochemistry; light gray, PHPT and underlying hyperplasia or adenoma; dark gray, PHPT and atypical adenoma; and black, PHPT and parathyroid carcinoma. +/+, Wild type (no deletion of *CDC73* gene exons 1–10); +/−, heterozygous deletion of *CDC73* gene exons 1–10; NA, not available; squares, male family members; circles, female family members; slash, deceased family member.

**Figure 2.**
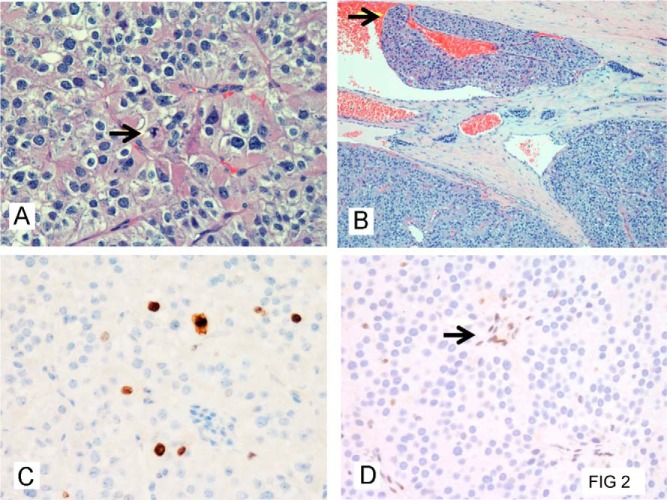
Parathyroid carcinoma. A, Monotonous growth of mainly chief cell-like tumor cells, with slide nuclear atypia. Arrow indicates mitotic figure (hematoxylin and eosin; magnification, ×400). B, Vascular invasion, arrow indicating tumor thrombus. C, Proliferation index (Ki67) of 5%, evaluated immunohistochemically by Mib-1 antibody. D, Negative parafibromin staining of neoplastic cells. Arrow indicates parafibromin-positive vascular endothelial cells that serve as an internal positive control.

Eleven members of a three-generation family with PHPT were ascertained (Figure 1 and Table 1). Informed written consent was obtained from all the patients. Clinical data from the two older generations as well as linkage to the HPT-JT locus on 1q21-q32 were reported previously (3). The affected relatives in the second generation shared haplotypes with the father (Figure 1, I-1) but not the mother (Figure 1, I-2) and thus had inherited the disease from the father (3). Both the father and the mother (Figure 1, I-1 and I-2) suffered from PHPT, but the father, who died in 2001 with a ruptured thoracic aneurysm at the age of 69 years, did not have parathyroid surgery. He was not willing to undergo regular screening for PHPT during his lifetime. His serum calcium was documented to be increased a year before he died. The mother (member I-2) was operated on for PHPT in 2011. Histopathology was compatible with parathyroid hyperplasia. Her serum ionized calcium has increased to preoperative concentrations and is currently 1.40 mmol/L (reference, 1.16–1.30 mmol/L). Plasma biochemistry using morning fasting samples was performed at the Helsinki University Central Hospital Laboratory using standard methods.

**Table 1.** Characteristics of Family Members

ID No.	Sex (F/M)	Year of Birth	PHPT (Age at Surgery, y)	Calcium Ion in Nonoperated Subjects, mmol/L	Histology	Renal Cysts	Aortic Aneurysm	*CDC73* Deletion
I-1	M	1932	Yes	NA		No	Yes	NA
I-2	F	1934	Yes (77)		HP	No	No	No
II-1	F	1953	No	1.24				No
II-2	M	1955	Yes (36)		A	No	No	Yes
II-3	F	1956	Yes (37)		AA	Yes	No	Yes
II-4	M	1959	Yes (38)		A	No	No	Yes
II-5	M	1961	Yes (32)		CA	Yes	Yes	Yes
III-1	F	1974	No	1.21				Yes
III-2	F	1978	Yes (31)		A	No		NA
III-3	M	1993	Yes (18)		CA, AA	No	No	Yes
III-4	F	1993	No	1.26				No
III-5	F	1987	No	1.30				Yes
III-6	F	1995	No	1.26				No

Abbreviations: F, female; M, male; A, parathyroid adenoma; AA, atypical adenoma; CA, parathyroid carcinoma; NA, not available; HP, hyperplasia. The reference range for serum calcium ion is 1.16–1.30 mmol/L.

### Multiplex ligation-dependent probe amplification (MPLA) analysis

Genomic DNA extracted from whole blood was used in an MLPA assay, which is a dosage-based technique for detection of deletions and duplications of one or more exons. An MLPA kit P200-A1 (MRC Holland) was used as a reference kit, with the addition of an in-house-designed synthetic probe mix to detect deletions or duplications of *CDC73.* This assay did not include probes for exons 5, 12, 14, or 16, but a probe for all other exons (1–4, 6–11, 13, 15, and 17) was included.

### Immunohistochemistry

Immunostaining was performed using deparaffinized tissue section utilizing mouse monoclonal Ki-67 antibody (clone MIB-1; Dako), 1:100 for proliferation index, as well as mouse monoclonal parafibromin antibody (clone sc-33638; Santa Cruz Biotechnology), 1:1000 with the polymer detection kit EnVision (Dako) in a LabVision Autostainer (Thermo Scientific); sections were counterstained with Mayer's Hematoxylin (Lillie's Modification) (Dako) and mounted with Mountex (Histolab). Ki-67 proliferation indices were assessed in 2000 neoplastic cells.

## Results

### *CDC73* gene analyses

MPLA analysis of the *CDC73* gene revealed a heterozygous deletion of exons 1–10 in seven of the available 11 family members (Figure 1 and Table 1). Five of these individuals with the *CDC73* deletion had been operated on for PHPT at the ages of 18–38 years (Figure 1 and Table 1), whereas the other two (Table 1, III-1 and III-5), aged 39 and 26 years at the last biochemical screening, did not have PHPT.

### Phenotypes of family members affected with PHPT

Family members II-5 and III-3 (Figure 1 and Table 1) presented with severe PHPT, headaches, and hypertension at the ages of 32 and 18 years, respectively. Blood pressure normalized in both subjects after primary parathyroidectomy. Family member II-5 was most severely affected. He was diagnosed with severe PHPT at age 32 years, the initial symptoms being headaches and hypertension (blood pressure values were 160–180/110–130 mm Hg). Surgery revealed a 3-cm encapsulated parathyroid carcinoma of the left lower parathyroid gland. The right parathyroid glands were macroscopically normal and were left intact. He had his first recurrence <2 years later when a 2-cm lymph node metastasis was resected from the left side of the neck. Recurrence was preceded by rising serum calcium and PTH concentrations and recurrent hypertension. A second neck exploration was performed at age 36 years, when an adenoma of the right upper parathyroid gland, a normal left lower parathyroid, and a lymph node metastasis were resected. He has since had more than nine operations because of recurrent hypercalcemia. Distal metastases located close to the spine were resected at ages 38, 39, and 49 years. Family member II-5 was also diagnosed with a thoracic aortic aneurysm, which was operated at the age of 51 years, and family member I-1 died at the age of 69 because of a ruptured thoracic aortic aneurysm (Figure 1 and Table 1).

Family member III-3 was diagnosed at the age of 18 years with severe hypertension (blood pressure, 210/110–115 mm Hg) and headaches. He had severe PHPT, with serum ionized calcium of 1.88 mmol/L (normal, 1.16–1.30 mmol/L) and serum PTH of 693 ng/L (normal, 8–73 ng/L). Investigations revealed that he did not have pheochromocytoma or primary hyperaldosteronism. Bilateral neck exploration was performed; a 1.5-cm enlarged upper right parathyroid was resected, and the right lower parathyroid gland was left intact. The left upper parathyroid was also enlarged at 1.4 cm and was resected, as well as the left lower parathyroid gland, which was macroscopically normal. Serum calcium ion normalized immediately postoperatively (from 1.30 to 1.19 mmol/L), and serum PTH was 10 ng/L on the first postoperative day. The headaches and hypertension resolved. He had carcinoma of the right upper parathyroid gland and atypical adenoma of the left upper gland. The left lower parathyroid was normal. Among the other affected family members, family member II-3 had an atypical adenoma (Table 1), whereas members II-2, II-4, and III-2 had parathyroid adenomas. Two family members (II-3 and II-5; Table 1) have bilateral renal cysts. X-ray did not reveal any jaw tumors in this family.

### Ki67 proliferation index and parafibromin stain in parathyroid carcinoma

Histopathological examination of the right upper gland of family member III-3 (18-year-old male; Figure 1 and Table 1) demonstrated parathyroid carcinoma with vascular invasion (Figure 2, A and B). The Ki-67 proliferation index was 5% (Figure 2C), and nuclear parafibromin immunostaining was negative (Figure 2D). The left upper parathyroid was an atypical adenoma that did not immunostain for parafibromin. In family member II-5 (Figure 1 and Table 1), the Ki-67 of the primary parathyroid carcinoma resected at age 32 years was 14.5%, and that of a neck lymph node metastasis resected at age 49 years was 20%. These parathyroid carcinoma and lymph node metastasis did not immunostain for parafibromin.

## Discussion

MPLA analysis revealed that a previously unreported heterozygous deletion of *CDC73* exons 1–10 is the cause of PHPT in this three-generation family, thereby highlighting the need for performing *CDC73* deletion analysis in parathyroid carcinoma patients, and FIHP and HPT-JT families who do not have point mutations of *CDC73*. This represents the fourth report of a *CDC73* deletion. Whole *CDC73* deletions have been previously reported in a 25-year-old Portuguese man with severe, sporadic PHPT due to a single parathyroid adenoma (17) and a Spanish HPT-JT family, in which the index patient was an 18-year-old female who had jaw tumors and PHPT due to an adenoma that stained negative for parafibromin (13). Analysis of 250 PHPT patients reported 7% (n = 20 patients) to have a germline *CDC73* abnormality; *CDC73* deletions were found in seven patients, with three having whole gene deletions and four having intragenic deletions involving exons 3, 2 and 3, 4–6, and 7–13, respectively (15).

The most severe PHPT and phenotypes were encountered in two males with the exon 1–10 deletion of *CDC73*, and both of these had parathyroid carcinoma, at ages 32 and 18 years; they also had headaches and severe hypertension as the initial manifestations of PHPT, which resolved after primary surgery. Hypertension is not a usual feature of PHPT; today, PHPT is often a biochemically mild, asymptomatic disorder, or it involves diffuse symptoms such as obstipation and fatigue (1). Genotype-phenotype correlations were not apparent within this family, consistent with previous reports (4, 5). Thus, deletion of *CDC73* exons 1–10 resulted in parathyroid carcinoma diagnosed at the ages of 18 and 32 years in two male family members (Table 1; members III-3 and II-5, respectively), whereas other members have parathyroid adenomas or atypical adenomas. In addition, two affected males had thoracic aortic aneurysms, which have not been previously reported to be associated with FIHP, HPT-JT, or *CDC73* mutations, and it is possible that aortic aneurysms may be a novel feature of these disorders. Alternatively, thoracic aneurysms represent another trait in this family unrelated to the *CDC73* deletion. Two mutation carriers (members III-1 and III-5; ages 39 and 26 y) have not yet developed PHPT. The older one has a serum ionized calcium of 1.21 mmol/L and may represent a case of incomplete penetrance. The younger one might still develop PHPT because her current serum calcium is 1.30 mmol/L, at the upper reference limit. There is not much data on unaffected mutation carriers in the literature. Guarnieri et al (18) reported a family with FIHP caused by a *CDC73* frameshift mutation, where three of 10 mutation carriers had either an atypical parathyroid adenoma (n = 2) or parathyroid carcinoma (n = 1), but only one of the mutation carriers was hypercalcemic. The authors concluded that longitudinal surveillance including neck ultrasound is important (18).

The parathyroid carcinomas and the atypical adenoma did not immunostain for parafibromin, and the Ki-67 proliferation indices of the parathyroid carcinomas were 5 and 14.5%. The aggressive and recurrent behavior of the parathyroid carcinoma is consistent with observations that indicate that the presence of a *CDC73* mutation and loss of parafibromin immunostaining predicts significantly higher recurrence rates and lower overall 10-year survival (17). Loss of parafibromin immunostaining has been reported to be a better predictor of clinical outcome and mortality rates than *CDC73* mutation (20), and our findings are consistent with these observations. Further studies are needed to evaluate the predictive value of these tumor markers (17, 19–21) in patients with *CDC73* germline mutations in sporadic cases of parathyroid carcinomas and atypical adenomas.

In summary, we report a previously unreported intragenic deletion involving exons 1–10 of the *CDC73* gene in familial PHPT associated with parathyroid carcinoma. In addition to parathyroid tumors, this deletion was associated in two males with thoracic aortic aneurysms, which possibly represents a new manifestation of *CDC73* mutations. The findings of the present study underline the need for performing *CDC73* deletion analysis in HPT-JT, FIHP, parathyroid carcinoma, and/or severe early-onset PHPT patients who do not have point mutations.
